# Supplementary material for: The influence of social capital in the utilisation of sexual reproductive health services among the youth in Ghana. A community-based cross-sectional study
Source: PLOS Glob Public Health. 2023 Oct 5;3(10):e0001225. doi: 10.1371/journal.pgph.0001225 (PMC10553252; doi:10.1371/journal.pgph.0001225)
Supplement: S1 Questionnaire — (PDF) [file pgph.0001225.s001.pdf]

APPENDIX

Appendix I: Survey Questionnaire

University of Ghana, School of Public Health
Project Title: The relationship between Social Capital, Risky Sexual Behaviour and Utilization of Reproductive Health Services among the Youth in South Tongu

My name is ______________________ and a researcher working for a PhD student at the University of Ghana, Legon. I will be grateful if you can share some 20 – 25 minutes of your time with me to discuss some issues that relates to the reproductive health of the youth in Ghana, and especially in this community. Some of the questions we will be dealing with concern issues of boyfriends/girlfriends and sexual behaviors. For some people, these topics are private matters and may be embarrassing to discuss. I appreciate any information you may be willing to share with me on these topics. I also want to remind you that you are free to skip any question that you do not want to answer. In fact, for our research, we would rather have you skip a question than give inaccurate information. So please, if you do not want to answer a question or feel that you cannot answer it honestly, please just ask me to skip ahead to the next question and I will be happy to do so.

(IF PARTICIPANT AGGREED TO TAKE PART IN THE STUDY, INTERVIWER SHOULD ASK THE FOLLOWING QUESTIONS)

If you agree to take part, may I know if you currently have a sexual partner (boyfriend/girlfriend)? (TERMINATE IF NOT), and also if you ever had sexual intercourse? (TERMINATE IF NOT).

Respondent ID: _____/_____/_____/_____<br><br>Date of interview: (dd/mm/yy)<br>_____/_____/_____	Name of research assistant: ……….<br><br>Location of Interview …………………..<br><br>Urbanisation<br>1. Urban

	2. Rural

I 'll like to start by asking you some basic questions about yourself, school and family.

SECTION A: SOCIO-DEMOGRAPIC DATA

**Question No.**	**Question**	**Response**
SDI 1	What is your sex	1. Male<br>2. Female
SDI 2	How old were you on your last birthday	___/___/ Years
SDI 3	Are you currently in School	1. Yes (Continue)<br>2. No (Skip to SDI 8)
SDI 4	In which class/form/level are you	Write as appropriate<br>____________________
SDI 5	What was your last level of Education	1. No formal education<br>2. Primary<br>3. JSS /JHS<br>4. SSS /SHS<br>5. Tertiary
SDI 6	24	1. No formal education<br>2. Primary<br>3. JSS /JHS<br>4. SSS /SHS<br>5. Tertiary
SDI 7	What is the highest level of education of your Mother?	1. No formal education<br>2. Primary

		3. JSS /JHS<br>4. SSS /SHS<br>5. Tertiary
SDI 8	What is your occupation?	Write as appropriate<br>____________________
SDI 9	What is your marital status	1. Single (Not Married)<br>2. Married<br>3. Co-habiting
SDI 10	Which ethnic group do you belong to?	1. Ewe<br>2. Ga/Dangme<br>3. Akan<br>4. Other (specify)…….......
SDI 11	What is your religion	1. None<br>2. Christianity<br>3. Muslim<br>4. Traditionalist<br>5. Others<br><br>(Specify)………………….
SDI 12	Whom have you been living with over the last 12 months	1. Both parents<br>2. Mother<br>3. Father<br>4. Grandparents<br>5. Sibling<br>6. Step Parents<br>7. Other family member<br>8. Guardian (non family)<br>9. Other (specify) ………………………..

SECTION B: INTEGRATED SOCIAL CAPITAL QUESTIONNAIRE

Groups and Networks

GN1. I'd like to start by asking you about the groups or organizations, networks, associations to which you or any member of your household belong. These could be formally organized groups or just groups of people who get together regularly to do an activity or talk about things. As I read the following list of groups, please tell me if anyone in this household belongs to such a group. If yes, tell me which household member is most active in this group, and whether he/she participates actively in the group's decision making.

Type of Organisation	Name or Organisation	How actively does this person participate in the group's decision making?<br>1 = Does not participate in decision making<br>2 = Somewhat Active<br>3 = Very Active<br>4 = Leader
A. Religious or spiritual group		
B. Political group or movement		
C. Cultural group or association (e.g. arts, music, theater, film)		

D. Finance, credit or savings group		
E. Health group		
F. Sports group		
G. Youth group		
H. NGO or civic group (e.g. Rotary Club, Red Cross)		
I. Other Groups		

GN2. Of all the groups to which you belong, which two are the most important to you?

[ENUMERATOR: WRITE DOWN NAMES OF GROUPS]

Group 1 ____

Group 2 ____

GN3. Thinking about the members of this group, are most of them of the same…

	Group 1	Group 2
A. Neighbourhood/Village		
B. Family or Kin group		
C. Religion		
D. Gender		
E. Age		
F. Ethnic or linguistic group/race/ caste/tribe		

GN4. Does this group work or interact with other groups with similar goals *in* the village/Neighbourhood?

Group 1

NO	Yes Occasionally	YES, Frequently
1	2	3

Group 2

NO	Yes Occasionally	YES, Frequently
1	2	3

GN5. About how many close friends do you have these days? These are people you feel at ease with, can talk to about private matters, or call on for help.

GN6. If you suddenly needed a small amount of money [RURAL: enough to pay for expenses for your household for one week; URBAN: equal to about one week's wages], how many people beyond your immediate household could you turn to who would be willing to provide this money?

No one	One or two people	Three or four people	Five or more people
0	1	2	3

GN7. If you suddenly faced a long-term emergency such as the death of a breadwinner or [RURAL: harvest failure; URBAN: job loss], how many people beyond your immediate household could you turn to who would be willing to assist you?

No one	One or two people	Three or four people	Five or more people
0	1	2	3

GN8. [IF NOT ZERO] Of those people, how many do you think are currently able to assist you?

Trust and Solidarity

In every community, some people get along with others and trust each other, while other people do not. Now, I would like to talk to you about trust and solidarity in your community.

TS1. Generally speaking, would you say that most people can be trusted?

NO, Most people cannot be trusted	YES, Most people can be trusted
0	1

TS2. In general, do you agree or disagree with the following statements?

	1. Disagree strongly<br>2. Disagree somewhat<br>3. Neither agree nor disagree<br>4. Agree somewhat<br>5. Agree strongly
A. Most people who live in this village/Neighbourhood can be trusted.	
B. In this village/Neighbourhood, one has to be alert or someone is likely to take advantage of you.	
C. Most people in this village/Neighbourhood are willing to help if you need it.	

D. In this village/Neighbourhood, people generally do not trust each other in matters of lending and borrowing money.	

TS3. Now I want to ask you how much you trust different types of people. On a scale of 1 to 5, where 1 means a very small extent and 5 means a very great extent, how much do you trust the people in that category?

	1. To a very small extent<br>2. To a small extent<br>3. Neither small nor great extent<br>4. To a great extent<br>5. To a very great extent
A. People from your ethnic or linguistic group/race/caste/tribe	
B. Local government officials	
C. Central government officials	
D. Teachers	
E. Nurses and doctors	

TS4. Do you think that over the last five years*, the level of trust in this village/Neighbourhood has gotten better, worse, or stayed about the same?

[* ENUMERATOR: TIME PERIOD CAN BE CLARIFIED BY SITUATING IT BEFORE/AFTER MAJOR EVENT]

Gotten better	Gotten worse	Stayed about the same
1	2	3

TS5. How well do people in your village/Neighbourhood help each other out these days? Use a five-point scale, where 1 means always helping and 5 means never helping.

Always helping	Helping most of the time	Helping sometimes	Rarely helping	Never helping
1	2	3	4	5

Information and Communication

IC1. How many times in the last month have you or anyone in your household read a newspaper or had one read to you?

IC2. How often do you listen to the radio?

Never	Less than once a week	Once a week	A few times a week	Every day
1	2	3	4	5

IC3. How often do you watch television?

Never	Less than once a week	Once a week	A few times a week	Every day
1	2	3	4	5

IC3. How often do you visit social media (Facebook, WhatsApp, twitter, Instagram etc)?

Never	Less than once a week	Once a week	A few times a week	Every day
1	2	3	4	5

IC5. What are the three most important sources of information about what the government is doing reproductive health (such as family planning, etc.)?

Relatives, friends and neighbors	
Community bulletin board	
Local market	
Community or local newspaper	
National newspaper	
Radio	
Television	
Groups or associations	
Business or work associates	
Political associates	
Community leaders	

An agent of the government	
NGOs	
Social Media (Facebook, WhatsApp, twitter, Instagram etc)	

IC5. In general, compared to five years ago*, has access to information improved, deteriorated, or stayed about the same? [* ENUMERATOR: TIME PERIOD CAN BE CLARIFIED BY SITUATING IT BEFORE/AFTER MAJOR EVENT]

Deteriorated	Stayed about the same	Improved
1	2	3

Social Cohesion and Inclusion

SCI 1. Do the majority of people in this area generally have good relationships with each other?

NO	Sometimes	YES
1	2	3

SCI 2. How strong is the feeling of togetherness or closeness in your village/neighbourhood? Use a five-point scale where 1 means feeling very distant and 5 means feeling very close.

Very distant	Somewhat distant	Neither distant nor close	Somewhat close	Very close
1	2	3	4	5

SCI 3. Do you feel that this area is yours?

NO	Sometimes	YES
1	2	3

SCI 4. There are often differences in characteristics between people living in the same village/neighbourhood. For example, differences in wealth, income, social status, ethnic background, or tribe. There can also be differences in religious or political beliefs, or there can be differences due to age or sex. To what extent do any such differences characterize your village/neighbourhood? Use a five-point scale where 1 means to a very great extent and 5 means to a very small extent.

To a very small extent	Neither great nor small extent	Neither great nor small extent	To a great extent	To a very great extent

1	2	3	4	5

SCI 5. How many times in the past month have you got together with people to have food or drinks, either in their home or in a public place?

SCI 6. Are there groups of people in the village/neighbourhood who are prevented from or do not have access to any of the following?

	1 Yes<br>2 No	How many are excluded?<br>1. Only a few people<br>2. Many people, but less than half of the village/neighbourhood<br>3. More than half the village/ neighbourhood
A. Education/schools		
B. Health services/clinics		
C. Water		
D. Justice		
E. Transportation		

SCI 7. Are there any community activities in which you are not allowed to participate?

Yes	No, I can participate in all activities ® skip to question SCI 8

SCI 8. In which activities are you not allowed to participate? [ENUMERATOR: LIST UP TO 3 ACTIVITIES

Empowerment

EM1. How much control do you feel you have in making decisions that affect your everyday activities? Do you have…

No control	Control over very few decisions	Control over some decisions	Control over most decisions	Control over all decisions
1	2	3	4	5

EM2. Do you feel that you have the power to make important decisions that change the course of your life? Rate yourself on a 1 to 5 scale, were 1 means being totally unable to change your life, and 5 means having full control over your life.

Totally unable to change life	Mostly unable to change life	Neither able nor unable	Mostly able to change life	Totally able to change life
1	2	3	4	5

EM3: Overall, how much impact do you think you have in making this village/neighbourhood a better place to live?

No impact	A small impact	A big impact
1	2	3

EM4: In the past 12 months, how often have people in this village/neighbourhood gotten together to jointly petition government officials or political leaders for something benefiting the community?

Never	Once	A few times (≤ 5)	Many times (> 5)
1	2	3	4

EM5: Were any of these petitions successful?

None were successful	Most were unsuccessful	Most were successful	Yes, all were successful
1	2	3	4

SECTION C

Sexual Behaviors that Contribute to Unintended Pregnancy and Sexually Transmitted Diseases, Including HIV Infection

No.	Question	Response

SB1	How old were you when you had sexual intercourse for the first time?	(Age in completed years)<br><br>……/……/
SB2	How many sexual partners (boyfriend/girlfriend) have you had in your lifetime?	……/……/
SB3	Within the last 12 months, how many sexual partners have you had?	……/……/
SB4	During the past 12 months, with how many people have you had sexual intercourse?	……/……/
SB5	The first time you had sexual intercourse, did you or your partner use a condom?	1. Yes<br>2. No
SB6	The last time you had sexual intercourse, did you or your partner use a condom?	1. Yes<br>2. No
SB7	During the past 12 months, have you had casual sex (that is with somebody who is not your boyfriend/girlfriend)	1. Yes<br>2. No
SB8	Within the last 12 months how many casual sex partners have you had?	……/……/
SB9	In the past 12 months have you pay to had sex with a commercial sex worker? Or have you been paid to have sex?	1. Yes<br>2. No
SB10	Within the last 12 months did you ever drink alcohol or use drugs before you had sexual intercourse	1. Yes<br>2. No
SB11	Have you dated someone who is 10 or more years older than you?	1. Yes<br>2. No

SB12	The last time you had sexual intercourse, what one method did you or your partner use to prevent pregnancy?	1. No method was used to prevent pregnancy
		2. Birth control pills
		3. Condoms
		4. Depo-Provera (or any injectable birth control), Nuva Ring (or any birth control ring),
		5. Implanon (or any implant), or any IUD<br>6. Withdrawal
		7. Others (Specify)…

SECTION D

UTILIZATION OF SEXUAL AND REPRODUCTIVE HEALTH SERVICES

Question No	Questions	Response
RHS1	Have you ever heard of any service that addresses the sexual health of adolescents and the young adults?	1. Yes (Continue)<br>2. No (Skip to RHS3)
RHS2	Which of these sexual health services do you know of? *INTERVIEWER TO TAKE FIRST THREE MENTION*	1. Family Planning
		2. Pregnancy testing
		3. Mother and child health (ANC, Postnatal services etc)
		4. Treatment of Sexually Transmitted Infections
		5. VCT Services (HIV/AIDS)
		6. Condom provision
		7. Pap smear (screening for cancer of the cervix)
		8. Other, specify _______________

RHS3	(PROMPT RESPONDENTS)<br><br>Have you heard about the following Youth Sexual and Reproductive Health services? Interviewer to read the rest of the services.	1. Family Planning
		2. Pregnancy testing
		3. Mother and child health (ANC, Postnatal services etc)
		4. Treatment of Sexually Transmitted Infections
		5. VCT Services (HIV/AIDS)
		6. Condom provision
		7. Pap smear (screening for cancer of the cervix)
		8. Other, specify _______________
RHS4	Where did you hear it from it?	1. School<br>2. Church<br>3. Social Media<br>4. Mass media<br>5. Friends<br>6. Family<br>7. Health worker<br>8. Others (Please specify) ……………………..
RHS5	Which of the services have you ever used?	Family Planning
		Pregnancy
		Mother and child health
		Treatment of Sexually Transmitted Infections
		VCT Services (HIV/AIDS)
		Condom provision

		Pap smear (screening for cancer of the cervix)
		Other, specify _______________
RHS6	Which of the services have you used in the last 12 months?	Family Planning
		Pregnancy
		Mother and child health
		Treatment of Sexually Transmitted Infections
		VCT Services (HIV/AIDS)
		Condom provision
		Pap smear (screening for cancer of the cervix)
		Other, specify _______________
RHS7	Where did you get these services? *You can tick more than one option*	1. Government health facility
		2. Private health facility
		3. Pharmacy/Chemist
		4. Other, specify
RHS8	Do you know about Adolescent/Youth Friendly Corners?	1. YES (CONTINUE)<br>2. NO (SKIP TO HSE 1)
RHS9	If 'yes' where did you get information about Adolescent/Youth Friendly Corners?	1. School<br>2. Church<br>3. Social Media (WhatsApp groups, Facebook, Twitter, Instagram)<br>4. Mass media (Radio, TV, magazines, Newspapers<br>5. Friends<br>6. Family

RHS10	Have you ever visited a Youth Friendly Service facility?	1. YES (CONTINUE)<br>2. NO (SKIP TO RHS 12 AND END INTERVIEW)
RHS11	How many times have you visited the youth friendly service facility in the past 12 months?	____/ ____ SKIP TO RHS13
RHS12	If you have never visited or used a Youth Friendly Service, why?	1. Do not know about it<br>2. Poor quality of services<br>3. Location of the YFS facility<br>4. Inconvenient hours of operation<br>5. Long waiting hours<br>6. Lack of privacy and confidentiality<br>7. Staff attitudes and behaviours<br>8. Poor facility (building) structures<br>9. Use of services is against my values and beliefs<br>10. Other _________________________
RHS 13	On a scale of 1 to 5 where 1 = 'not satisfied at all' and 5 = 'very satisfied', how will you rate your level of satisfaction on the following attributes:<br>a. The quality of services provided at the facility<br>b. The level of professionalism exhibited by service provider<br>c. The cost of the services provided<br>d. Location of service provider<br>e. Privacy	1. Not satisfied at all<br>2. Not satisfied<br>3. Somewhat satisfied<br>4. Satisfied<br>5. Very satisfied

HEALTH SYSTEM AND ENVIRONMENTAL FACTORS		
HSE 1	Do you have a valid NHIS Card?	1. YES<br>2. NO
HSE 2	Approximately how long does it take you to get to the nearest health facility	Write as appropriate<br>…………………..
HSE 3	What is the mode of transport to the nearest health facility	Write as appropriate<br>…………………..
