# Supplementary material for: The influence of social capital in the utilisation of sexual reproductive health services among the youth in Ghana. A community-based cross-sectional study
Source: PLOS Glob Public Health. 2023 Oct 5;3(10):e0001225. doi: 10.1371/journal.pgph.0001225 (PMC10553252; doi:10.1371/journal.pgph.0001225)
Supplement: Attachment — Submitted filename: Response to Reviewers.docx [file pgph.0001225.s003.docx]

Mark Kwame Ananga

Department of Population and Behavioural Sciences

Fred N Binka School of Public Health

University of Health and Allied Sciences

26th February, 2023

Dear Editor

I write to submit a revised manuscript on the role of social capital in reproductive health service utilisation.

Kindly find below the various comments and our response to each of the comments.

**REVIEWER 1**

COMMENT: *This is cross-sectional study and causation cannot be determined. Therefore, the paper should avoid causal language that appears in several places, such as in Abstract “led to increased use…”.*

RESPONSE: All causal statements have been removed. For instance, “Access to some social capital dimensions *led to increased use* of reproductive health services” has been change to “Access to some social capital dimensions ***is associated with increased use*** of reproductive health services”. ***See page 1 of manuscript with track changes***

COMMENT: *The reader would need to know at least 1) what questions were used for which indicators, 2) exactly how the variables were combined to create a score and 3) the cut-offs used to categorize into low and high levels for each social capital variable.*

RESPONSE: The section 2.5 Study variables has been rewritten to cater for the questions used and how variables were combined to create a score. ***See pages 5 – 7*** ***of manuscript with track changes***

COMMENT*:* *In addition, the questionnaire should be available, at least as a supplementary file*

RESPONSE: Questions are now available as a supplementary file

COMMENT: *Results: The text repeats what’s shown in the tables. Please avoid redundancy by trimming the text.*

RESPONSE: Results section has been revised to meet the requirements of the reviewer

COMMENT: *Tables 2 and 3. Please provide a rationale for the stratification by sex and rural residence in the methods*.

RESPONSE: Rationale for stratification was provided. Section 2.8. Data Analysis and Presentation, ***See page 8 -9 of manuscript with track changes***

COMMENT: *Figure 1 is not needed since this information can easily be included in Table 3*

RESPONSE: Fig 1 has been removed.

COMMENT: *It is not clear how the modelling strategy and choice of covariates were determined.*

RESPONSE: The strategy for the modelling and choice of covariates has been addressed in Section 2.8. Data Analysis and Presentation, ***See page 9 of manuscript with track changes***

COMMENT*: Abstract refers to adjustment for sex, marital status, age and access to valid National Health Insurance Card but the footnotes of Table 4 suggest more variables were included.*

RESPONSE: Abstract has been reviewed to read *“after controlling for other significant predictors”.* ***See page 1 of manuscript with track changes***

The data analysis section in the methods is very general and does not provide enough details.

COMMENT: *Residual confounding should be noted in the limitations.*

RESPONSE: Residual confounders have been noted as a limitation. ***See page 26*** ***of manuscript with track changes***

**REVIEWER 2**

COMMENT: First the article analyses social capital in the frame of the diverse obstacles which persist in accessing youth friendly SRH services. Improving on young people's social capital is presented as an alternative policy option, which can make up for service failure and stigma in the community. This proposition was trendy for a while at the beginning of the years 2000, in particular in World Bank publications, but never gained momentum. Scarce funds for youth SRH services are indeed probably better invested elsewhere than in building individuals' social capital.

*I thus strongly suggest to focus the article* ****only**** *on social capital as a factor of access to youth SRH services.*

RESPONSE: Thank you for that valuable comment. This article is part of larger PhD dataset. This particular article focussed on only one district that have access to reproductive health services. In the South Tongu District, there are the Plan Parenthood Association of Ghana , that is very visible and vibrant with reproductive health services for young people, a District Hospital with a vibrant Adolescent and Youth Corner, a as well smaller Health facilities that provide reproductive health services to the youth. However, the utilisation of these services over the years have been very low. Therefore, this particular article is solely about utilisation and not access.

The comments are however, valuable to be incorporated in the PhD Thesis.

*COMMENT: Some of the items like "empowerment" seem fully out of the scope of what is usually meant by social capital.*

RESPONSE: Empowerment was included in this analysis because social capital contributes to empowerment process. Adolescent and youth reproductive health services utilisation cannot be isolated from the effect of empowerment on the individual.

*COMMENT: The questionnaire used in this study, which dates back to 2004 (World Bank) may be partly outdated. The exact way in which the dimension is measured (the wording of the question) should be spelled out. The compound indicator "overall social capital" is also probably to be abandoned, for the same reason.*

RESPONSE: This has been revised in Section 2.5. ***See pages 5 – 7 of manuscript with track changes.*** In addition, the questionnaire has been addended as a supplementary material. Further, the compound indicator “overall social capital” has been removed from the Table.

*COMMENT: First, why were age and sex, and other important covariates, not investigated and controlled for in relation to social capital?*

RESPONSES: All important covariates were investigated. Preliminary analysis shows that ***only Sex and Area of Residence had significant relationship with access to social capital***. The preliminary findings formed the basis for the stratification of all the results on sex and area of residence

*COMMENT: Second, the notes a, b and c .. in the multivariate analyses tables are not understandable.*

RESPONSES: Explanation provided. See Section 2.8, pages 8-9 ***of manuscript with track changes***.

*COMMENT: Also show the size of the effects for all the covariates, as a recurrent finding in the social capital health literature is that effect sizes are small.*

RESPONSE: Effects size for all covariates has been calculated. The results incorporated into Table 4 and Table 5
