# Supplementary material for: The influence of social capital in the utilisation of sexual reproductive health services among the youth in Ghana. A community-based cross-sectional study
Source: PLOS Glob Public Health. 2023 Oct 5;3(10):e0001225. doi: 10.1371/journal.pgph.0001225 (PMC10553252; doi:10.1371/journal.pgph.0001225)
Supplement: Attachment — Submitted filename: Response to Reviewers2.docx [file pgph.0001225.s004.docx]

Mark Kwame Ananga

Department of Population and Behavioural Sciences

Fred N Binka School of Public Health

University of Health and Allied Sciences

24^th^ June, 2023

Dear Editor

I write to submit a revised manuscript on the role of social capital in reproductive health service utilisation.

Kindly find below the various comments and our response to each of the comments.

**REVIEWER 3**

COMMENT *1. On the abstract, background section, stated that “Besides, while the factors associated with the use of reproductive health services among the youth are well documented in the literature, most studies have focused on aspects at the individual level.” It is good to examine the overlooked factors, but, the concept of social capital is not only at the societal level. Instead, it comprises at individual, community, institution levels.…”.*

RESPONSE: Comment has been noted and the sentence revised to ***read “Besides, while the factors associated with the use of reproductive health services among the youth are well documented in the literature, most studies have focused on narrowed perspective failing to take cognisance of the role of social capital”.*** See page 1

COMMENT *2. On the abstract, conclusion section stated that “any intervention meant to address social capital needs in sexual and reproductive health should consider the efficacy of each social capital dimension and the intervention's environment.” What types of interventions are appropriate for youths to address their social capital needs and be specific!*

RESPONSE: Paragraph has been revised to include the specific interventions. Thus, it now reads ***“However, any intervention such as mentoring including peer support programs, meant to address social capital needs in sexual and reproductive health should consider the efficacy of each social capital dimension and the intervention's environment”***. See page 1

COMMENT *3* *You mentioned the reason why you select the study population. Please cite the reference that as evidence. “This age group was chosen because most youth within this group are currently sexually active. (Reference?)”*

RESPONSE: Reference has been added. See page 4

COMMENT 4: *We cannot measure social capital directly because it is a latent variable. Why not you consider factor analysis that was appropriate to the variable?*

RESPONSE: Thank you for the valuable suggestion. We will still like to maintain the regression analysis. Factor analysis, while valuable for exploring the underlying structure of social capital, may not capture the full complexity of real-world social interactions. Regression analysis, on the other hand, allows you to examine the relationships between social capital and a broader range of variables, including socio-demographics and the dependent variable (use of SRH services). This enhances the external validity of our findings and provides a more comprehensive understanding of social capital and its relationship with the use of reproductive health services.

COMMENT 5: *Even though you have used a social capital questionnaire that was used in low-income setting, the questionnaire might not be comprehensive enough to consider the context of Ghana. In addition, the tool was not validated in the study area and should be mentioned as a limitation of your study.*

RESPONSE: Comment well noted. Limitations have been duly acknowledged. See page 22
